# Metabolic Profile and Evaluation of Biological Activities of Extracts from the Stems of *Cissus trifoliata*

**DOI:** 10.3390/ijms21030930

**Published:** 2020-01-31

**Authors:** Luis Fernando Méndez-López, Elvira Garza-González, María Yolanda Ríos, M. Ángeles Ramírez-Cisneros, Laura Alvarez, Leticia González-Maya, Jessica N. Sánchez-Carranza, María del Rayo Camacho-Corona

**Affiliations:** 1 Facultad de Ciencias Químicas, Universidad Autónoma de Nuevo León, Av. Universidad S/N Ciudad Universitaria, San Nicolás de los Garza C.P. 66451, Nuevo León, Mexico; luis.mendezl@uanl.mx; 2 Servicio de Gastroenterología Hospital Universitario Dr. José Eleuterio González, Universidad Autónoma de Nuevo León, Av. Gonzalitos y Madero S/N, Col. Mitras Centro, Monterrey C.P. 64460, Nuevo León, Mexico; elvira_garza_gzz@yahoo.com; 3 Centro de Investigaciones Químicas IICBA, Universidad Autónoma del Estado de Morelos, Av. Universidad 1001, Col. Chamilpa, Cuernavaca C.P. 62209, Morelos, Mexico; myolanda@uaem.mx (M.Y.R.); angelesrc@uaem.mx (M.Á.R.-C.); lalvarez@uaem.mx (L.A.); 4 Facultad de Farmacia, Universidad Autónoma del Estado de Morelos, Av. Universidad 1001, Col. Chamilpa, Cuernavaca C.P. 62209, Morelos, Mexico; letymaya@uaem.mx (L.G.-M.); jez_chazaq@hotmail.com (J.N.S.-C.)

**Keywords:** Hierba del buey, anticancer, GC-MS, LC-MS, bioactive compounds

## Abstract

*Cissus trifoliata* (L.) L belongs to the Vitaceae family and is an important medicinal plant used in Mexico for the management of infectious diseases and tumors. The present study aimed to evaluate the metabolic profile of the stems of *C. trifoliata* and to correlate the results with their antibacterial and cytotoxic activities. The hexane extract was analyzed using gas chromatography coupled with mass spectrometry (GC-MS) and the CHCl_3_-MeOH and aqueous extracts by ultraperformance liquid chromatography quadrupole time of fly mass spectrometry (UPLC-QTOF-MS). The antibacterial activity was determined by broth microdilution and the cytotoxicity was evaluated using MTS cell proliferation assay. Forty-six metabolites were putatively identified from the three extracts. Overall, terpenes, flavonoids and stilbenes characterize the metabolic profile. No antibacterial activity was found in any extract against the fifteen bacteria strains tested (MIC >500 µg/mL). However, high cytotoxic activity (IC_50_ ≤ 30 µg/mL) was found in the hexane and aqueous extracts against hepatocarcinoma and breast cancer cells (Hep3B, HepG2 and MCF7). This is the first report of the bioactive compounds of *C. trifoliata* stems and their antibacterial and cytotoxic properties. The metabolic profile rich in anticancer compounds correlate with the cytotoxic activity of the extracts from the stems of *C. trifoliata*. This study shows the antitumor effects of this plant used in the traditional medicine and justifies further research of its anticancer activity.

## 1. Introduction

Plants from the genus *Cissus* have been used globally in traditional medicine for the treatment of several diseases such as arthritis, obesity, cancer, infections and diabetes [1]. *Cissus* plants have shown a wide spectrum of medicinal properties, including anti-microbial [2], anti-inflammatory [3], anti-tumor [4], and anti-diabetic [5]. *Cissus* species produce a wealth of phytochemicals, including triterpenes, fatty acids, glycerolipids, steroids, phytols, cerebrosides, flavonoids and stilbenes [6,7]. The full bioactive compounds of these plants have yet to be elucidated, but among the bioactive phytochemicals isolated from *Cissus* plants are β-sitosterol, stigmasterol, ursolic acid, kaempferol, quercetin, resveratrol, and lupeol [8,9]. *Cissus trifoliata* (L.) L, also known as “Hierba del buey”, is a plant widely distributed in Mexico, Southern United States and the Caribbean South America. In Mexican traditional medicine, a decoction of its stems is applied to the affected site or used as infusion for the management of gastrointestinal illnesses [10] and tumors [11]. To our knowledge, there are no previous chemical studies of *C. trifoliata* or its antibacterial and cytotoxic activities. Currently, only one study has been carried out concerning the anti-inflammatory activity of extracts using murine models [12]. However, in vitro antibacterial activity of stem extracts from *C. quadrangularis* [13] and *C. pallida* [14] has been reported against *Bacillus subtilis*, *Klebsiella pneumoniae*, *Staphylococcus aureus*, *Escherichia coli*, *Salmonella typhi*, *Bacillus cereus* and *Pseudomonas aeruginosa*. On the other hand, cytotoxic effects of the stem extracts of *C. quadrangularis* [15], *C. verticillate* [16], *C. sicyoides* [17], and *C. debilis* [18] have been shown against HeLa, A431, HepG2 and CaCo-2 cells. Therefore, the ethnomedical knowledge and the chemotaxonomic relationship of *C. trifoliata* suggest that their stems might be a good source of bioactive compounds.

Metabolic profiling has been previously useful to understand the chemical diversity of a medicinal plant. This information can be used to compare it with taxonomically related studied plants and to infer their bioactivity [19,20,21]. Chromatography coupled to mass spectrometry is the most widely applied technology used for the analysis of samples in very complex matrices such as those of plant extracts [22]. The aim of this study was to investigate the metabolic profile of the hexane, CHCl_3_-MeOH and aqueous extracts of *C. trifoliata* stems by a GC-MS and Ultraperformance Liquid Chromatography-Quadrupole Time of Fly-Mass Spectrometry (UPLC-QTOF-MS) analysis. For their profiling, a database of reported compounds of the plants of the genus was established and used in conjunction with the available libraries. Based on high-mass accuracy, spectral data and previous reports, a tentatively compound identification was assigned. In order to shed more light on the medicinal use of *C. trifoliata*, the antibacterial and the cytotoxic activity were evaluated by microdilution and MTS assays. Additionally, the potential mechanism of action of the extracts was discussed according to their bioactive compound content.

## 2. Results

### 2.1. GC-MS Analysis of Hexane Stem Extract of C. trifoliata

**Figure 1:**
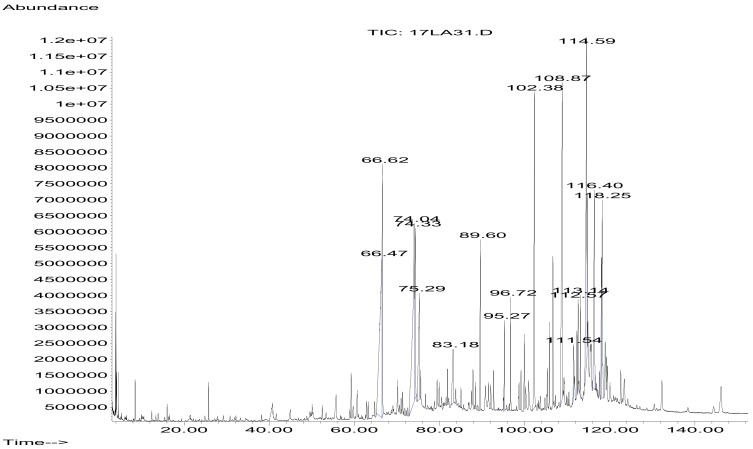
GC-MS chromatogram of hexane stem extract of *C. trifoliata*.

The volatile contents of hexane stem extract of *C. trifoliata* were analyzed by GC-MS. The chromatogram is showed in Figure 1. The identification of the components was based on the comparison of their GC-MS spectra and Kovats retention index with referent compounds in the NIST library [23] (Table 1). The hexane extract contained sixteen compounds belonging to the chemical classes of alkanes (18.7%), fatty acids (31.3%), terpenes (37.5%), alcohols (6.25%) and esters (6.25%).

### 2.2. UPLC-QTOF-MS Analysis of CHCl_3_-MeOH Stems Extract of C. trifoliata

**Figure 2:**
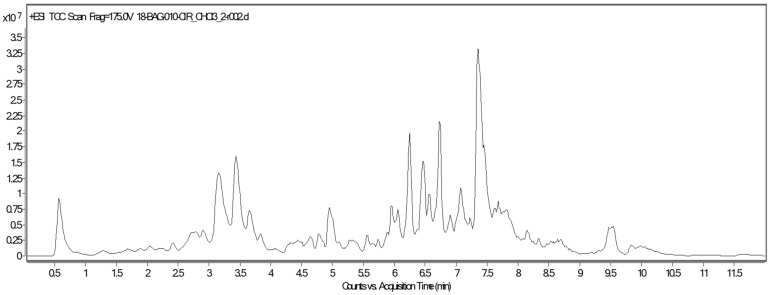
Chromatogram of UPLC-QTOF-MS analysis of CHCl_3_-MeOH stems extract of *C. trifoliata*.

The chromatogram of the UPLC-QTOF-MS analysis of CHCl_3_-MeOH stem extract of *C. trifoliata* is shown in Figure 2. Eighteen compounds were tentatively identified based on accurate *m*/*z* and the molecular formula [24] (Table 2). These included simple phenolics (16.6%), fatty acids (22.2%), flavonoids (44.6%), and stilbenes (16.6%).

### 2.3. UPLC-QTOF-MS Analysis of Aqueous Stems Extract of C. trifoliata

**Figure 3:**
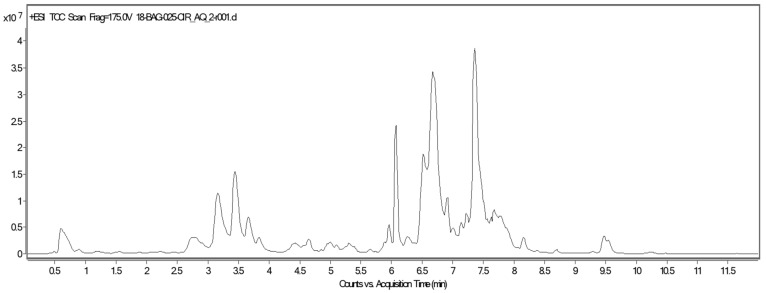
Chromatogram of UPLC-QTOF-MS analysis of aqueous stems extract of *C. trifoliata*.

The chromatogram of UPLC-QTOF-MS analysis of aqueous stem extract of *C. trifoliata* is shown in Figure 3. Twelve compounds were tentatively identified based on accurate *m*/*z* and the molecular formula [24] (Table 3). These include flavonoids (83%) and stilbenes (17%).

### 2.4. Biological Evaluation of C. trifoliata Stem Extracts

#### 2.4.1. Antibacterial Activity

Extracts were evaluated for their activity against fifteen bacteria, including sensitive and antibiotic-resistant strains. The antibacterial activity of the three *C. trifoliata* stem extracts was null against all the bacteria at the concentrations tested (500, 250, 125, 62.5, 31.2, 15.6 and 7.8 µg/mL). According to recommendations, plant extracts should exhibit antibacterial activity at MICs ≤ 30 µg/mL [25]. On the other hand, sensitivity to the positive control levofloxacin differs among the tested strains and showed inhibitory concentrations in the range of 3.12 to 50.0 µg/mL (Table 4).

#### 2.4.2. Cytotoxic Activity

The potential cytotoxic activity of *C. trifoliata* stem extracts was evaluated against six cancer cell lines: liver cancer (HepG2, Hep3B), breast cancer (MCF7), prostate cancer (PC3), cervix cancer (HeLa), and lung cancer (A549), that were selected because they represent the most studied cell models of the most common cancer types diagnosed in the Mexican population. Cancer cells were treated with extracts at concentrations of 100, 10, 1, 0.1, 0.001 µg/mL for a dose-response evaluation with an exposition of 72 h according to the literature [26]. The half maximal inhibitory concentration (IC_50_) was calculated for extracts and paclitaxel using MTS assays (Table 5). According to the criteria of the National Cancer Institute (NCI), extracts with IC_50_ ≤ 30 µg/mL should be considered cytotoxic [26]. Therefore, the results indicate that liver and breast cancer cells were more sensitive to the hexane extract and that breast cancer cells were more affected by the aqueous extract.

## 3. Discussion

### 3.1. Metabolic Profile of Stems Extracts from C. trifoliata

Metabolic profiling of plant extracts refers to the analysis by hyphenated techniques such as GC-MS and LC-MS [27]. Accurate mass spectrometry and spectral data are then processing with specific algorithms which provide a specific molecular formula and then the metabolites are identified in available databases [24]. Following this approach, forty-six metabolites were identified. The metabolic profiles of extracts from the stems of *C. trifoliata* included alcohols, alkanes, esters, fatty acids, terpenes and phenolic compounds. Overall, the compounds identified in the hexane extract are common constituents of cuticles and membranes of most plants [28,29]. The medicinal plant from the genus with most chemical and pharmacological studies is *C. quadrangularis*. The palmitic, stearic, linoleic and oleic fatty acids have been previously identified from its hexane stem extracts [30,31]. The terpenes squalene, beta sitosterol, campesterol, stigmasterol and lupeol have also been previously reported in the hexane and methanol extracts from the stems of *C. quadrangularis* [6,30,32].

On the other hand, most of the compounds identified by LC-MS were polyphenols. Flavonoids were the main chemical class identified, and kaempferol and quercetin glucosides account for most of them. Apigenin, kaempferol and quercetin have been reported on alcoholic extracts from *C. ibuensis* [9], *C. digitata* [33] and *C. quadrangularis* [34]. Stilbenes were the second most common class of polyphenolic compounds identified in the stems of *C. trifoliata*. Previously, resveratrol, piceatannol, and pallidol were isolated and characterized in ethanolic extracts from the stems of *C. quadrangularis* [8]. Stilbene glucosides have also been found in *C. repens* [35] and *C. sicyoides* [7].

In addition to previous reports of the phytochemical content of *Cissus* plants, their phylogeny also supports a metabolic profile characterized for a high content of flavonoids and stilbenes. For example, based on plastid markers, *Cissus* plants are genetically related with *Vitis* plants [36], the metabolomic profile of which showed overrepresentation of flavonoid and stilbene metabolites and their biosynthetic pathways [37]. Additionally, stilbene derivatives characterize and accumulate in the lignified stem tissue of Vitaceae plants [38].

### 3.2. Antibacterial Activity

The antibacterial activity of various extracts from the stems of *C. quadrangularis* has already been reported. The ethyl acetate, methanol and aqueous extracts showed inhibitory activity against the Gram-positive bacteria *Bacillus subtilis*, *Bacillus cereus*, *S. aureus* and *Streptococcus* species. In contrast, negative activity was found against the Gram-negative bacteria *E. coli* and *P. aeruginosa* [2]. Our study found null antibacterial activity against the fifteen bacteria tested. This may be explained because the antibacterial activity of *C. quadrangularis* was carried out by the agar well diffusion method. Additionally, the lowest concentration of extracts employed in the assays was 1000 µg/mL [2]. The highest concentration used in this study was 500 µg/mL in the micro-dilution broth method in 96-well microplates and extracts were considered inactive if the calculated MIC results were >30 µg/mL, according to recommendations of the National Committee for Clinical Laboratory Standards [25]. Thus, the results of the antibacterial activity analysis suggest that *C. trifoliata* extracts are inactive against bacteria although antibacterial properties against other strains or other antimicrobial activities cannot be excluded.

### 3.3. Cytotoxic Activity

The assessment of cytotoxicity of *C. trifoliata* extracts using MTS assay demonstrated activity against the six carcinoma cell lines exhibiting an IC_50_ values from 24 to 94 µg/mL. The extracts from the stems of *C. trifoliata* present potent activities against liver and breast cancer cells. The hexane extract was able to inhibit the proliferation of liver and breast cancer cells at 24-30 µg/mL, whereas the aqueous extract showed activity against breast cancer cells at 30 µg/mL. According to the National Cancer Institute plant, extracts with an IC_50_ ≤ 30 µg/mL possess good cytotoxic properties [26]. Previous reports in the literature demonstrated the cytotoxic activity of extracts from the stems of *Cissus* plants against cancer cell lines. The hexane and acetone extracts from *C. quadrangularis* showed cytotoxic activity against HepG2 and Hela cells (IC_50_ from 43-200 µg/mL). In another study, the ethyl acetate extract from *C. sicyoides* was cytotoxic for the HepG2 cells (IC_50_ of 50 µg/mL).

The cytotoxic or antiproliferative activity of *C. trifoliata* extracts may be mediated by their terpene, flavonoid and stilbene content. For example, stigmasterol showed cytotoxic activity against MCF7 cells (IC_50_ 14 µg/mL) [39]. β-Sitosterol also demonstrated cytotoxic activity against MCF7 (IC_50_ 8 µg/mL) [40] and Hep3B cells (IC_50_ 25 µg/mL) [41]. β-Sitosterol induces apoptosis mediated by caspase-8 activity [42] and by modulation of the estrogen receptor (ER), which inhibits the proliferation of sensitive cancer cells such as MCF7 [43]. Lupeol also possessed cytotoxicity activity against MCF7 (IC_50_ 32 µg/mL) and HepG2 cells (IC_50_ 48 µg/mL) [44]. Its mechanism of action was the induction of apoptosis through the mitochondrial cell death pathway and cell cycle arrest by inhibition of bcl-2 (B-cell lymphoma 2 protein) and CDKs (cyclin-dependent kinases) [45]. The polyphenols resveratrol, quercetin and kaempferol have been showed several anticancer mechanisms of action. For example, resveratrol induces cell-cycle arrest and acts as anti-estrogen in MCF7 cells (IC_50_ 32 µg/mL) [46]. Kaempferol also blocks the cell cycle and ER signaling acting. Doses of 50-100 μM decreased the cell viability in MCF7 and downregulated the expressions of cyclin proteins D1 and E, but increased p21 protein expression (p21 Cyclin-dependent kinase inhibitor) [47]. Quercetin showed similar mechanisms of action in MCF cells (IC_50_ 50 μM/mL), inhibited the proliferation and induced apoptosis by increasing caspase-3 expression [48]. Additionally, quercetin also possessed ER antagonism [49]. Together, these studies suggest synergistic activity of the bioactive compounds of the extracts of *C. trifoliata* against cancer cells.

Gross metabolic profiling has been previously useful to understand the bioactive content of medicinal plants, to compare it with taxonomically related studied plants and to infer or understand their bioactivity [19,20]. Accordingly, a metabolic profile high in terpenes, flavonoids and stilbenes in extracts from the stems of *C. trifoliata* was consistent with other studies of *Cissus* plants [8] and well characterized Vitaceae [37]. Moreover, the anti-tumor activity of *C. sicyoides* extracts in vivo has been attributed to β-sitosterol, quercetin, kaempferol and resveratrol [4]. Nonetheless, since a comparison with literature and databases was used for compound identification, to characterize at higher level of confidence, the inclusion of authentic standards is required [21]. Furthermore, since cytotoxic activity of the plant extracts was found against liver and breast cancer cells, it will be necessary to carry out a bio-assay guided study to isolate and characterize the bioactive compounds and to evaluate their mechanism of action in order to provide further understanding of the medicinal effects on this plant against tumors.

## 4. Materials and Methods

### 4.1. Plant Material and Extraction

*C. trifoliata* was collected and identified by a trained Biologist in Rayones, Nuevo León, Mexico (Latitude, 25.0167°, Longitude: −100.05°, Altitude: 900 m) on 10 October 2016. A voucher (027499) specimen was deposited in the Department of Botany of Universidad Autonóma de Nuevo León. The plant name has been checked in the website http://www.theplantlist.org. Dried and ground stems (756 g) were subjected to exhaustive extractions by maceration with hexane (4 L, 48 h), CHCl_3_-MeOH (1:1) (9 L, 4 times, 24 h each), and distilled water (4 L, 24 h). Solvents used were chloroform (CHCl_3_) purity 98.8%, methanol (MeOH) purity 99.9%, and hexane purity 98.99% (Baker, Phillipsburg, New Jersey, USA). The organic extracts were filtered and concentrated using a rotary evaporator at 40 °C (V300, Buchi, Flawil, Switzerland), and the aqueous extract was lyophilized. The extract yield was 3.5g (0.423%) for hexane, 24.g (3.201%) for CHCl_3_-MeOH, and 8.2g (1.084%) for aqueous. The dried extracts were kept at 4 °C until used.

### 4.2. GC-MS Analysis

The hexane extract was examined by GC-MS Agilent GC 6890, MSD 5973N (Agilent Technologies, Santa Clara, CA, USA) to determine its chemical composition. The analysis was conducted with the column HP-5MS (30 mm × 0.25 mm × 0.25 µm). The carrier gas was helium with a gas flow rate of 1mL/min and a linear velocity of 37cm/s. The injector temperature was set at 270 °C. The initial oven temperature was 70 °C and increased to 200 °C at 10 °C/min, 200 °C to 310 °C at 10 °C/min and the final temperature was held for 5 min at 310°C. The mass spectrometer was operated in the electron ionization mode at 70 eV and electron multiplier voltage at 1859 V. The retention index of compounds was recorded with standard n-hydrocarbon calibration mixture (C10-C40, Honeywell Fluka, Germany) using 2.64 AMDIS software. The compounds were identified by comparison of spectral data, fragmentation patter, and Kovats retention index with referent compounds in the NIST 17 database [23].

### 4.3. UPLC-QTOF-MS Analysis

Samples were diluted in LCMS grade MeOH (50%) (Fisher Scientific, Ottawa, Canada), filtered using Supelco (54145-U) Iso-disc, N-4-2 nylon, 4 mm × 0.2 µm filters (Fisher Scientific, Ottawa, Canada), and transferred to a high-recovery amber vial (Agilent Technologies, Santa Clara, CA, USA). Reverse-phase liquid chromatography was performed using an Agilent 1290 Infinity Ultra High-Performance Liquid The chromatography system (UHPLC) and the column ZORBAX C18, 2.1 × 50 mm, 1.8 µm (Agilent Technologies, Santa Clara, CA, USA) maintained at an isothermal temperature of 38 °C. The mobile phase was delivered by a binary pump at flow rate of 0.250 mL/min in a gradient elution using two mobile phases: LCMS grade water + 0.1% *v*/*v* formic acid (solvent A) (Fisher Scientific, Ottawa, Canada), and LCMS grade MeOH + 0.1% *v*/*v* formic acid (solvent B) (Fisher Scientific, Ottawa, Canada), with the following gradient conditions: 0-6 min, 100% solvent B; held at 100% 10 min, 100% B; 11 min, 30% B. The autosampler was set with an injection volume of 5 µL. The flush port was set to clean injection needle for 30s intervals. A mass spectrometric analysis was performed using an Agilent 6530 Quadrupole Time of Flight (QTOF) LCMS with an electrospray ionization (ESI) source (Agilent Technologies, Santa Clara, CA, USA). A mass spectrometry analysis was conducted in positive ion mode, set for a detection of mass-to-charge ratio (*m*/*z*) of 100 to 1000. The nebulizer pressure was set at 35 psi with a surrounding sheath gas temperature of 350 °C and a gas flow rate of 11 L/min. The drying gas temperature was set at 300 °C with a flow rate of 10 L/min. Default settings were used to set voltage gradient for the nozzle at 1000 V, skimmer at 65 V, capillary (VCap) at 3500 V, and fragmentor at 175 V. A record of LCMS data was taken using a MassHunter 6200 series TOF/6500 series Q-TOF B.05.01. MS acquisition was performed with three replicate injections to allow column conditioning and to examine reproducibility. Mass spectra were processed using the METLIN Database add-in for Agilent MassHunter Qualitative Analysis B.06.00. To putative compound identification, the correct elemental composition was generated using the accurate *m*/*z* and the molecular formula generation software (Agilent Technologies, Santa Clara, CA, USA) [24]. Data were queried against the online METLIN [24] and HMDB [50] databases.

### 4.4. Antibacterial Activity

The tested bacteria include seven bacteria from the ATCC (American Type Culture Collection, Manassas, VA, USA) and nine resistant strains isolated in the University Hospital of the Universidad Autonoma de Nuevo León (Monterrey, Nuevo León, Mexico). The bacteria from the ATCC include three gram-positive bacteria; *Staphylococcus aureus* (ATCC, 29213), *Staphylococcus epidermidis* (ATCC,14990) and *Enterococcus faecium* (ATCC, 2127) and four Gram-negative bacteria, *Acinetobacter baumanni* (ATCC, 13883), *Escherichia coli* (ATCC, 25922), *Pseudomonas aeruginosa* (ATCC, 27853), and *Klebsiella pneumoniae* (ATCC, 19606). The drug-resistant Gram-positive bacteria were methicillin-resistant *S. aureus* (14-2095), linezolid-resistant *S. epidermidis* (14-583), and vancomycin-resistant *E. faecium* (10-984). The drug resistant Gram-negative bacteria were carbapenem-resistant *A. baumannii* (12-666), extended spectrum β-lactamase (ESBL) *E. coli* (14-2081), carbapenem-resistant *P. aeruginosa* (13-1391), oxacillin-resistant (OXA-48) *K. pneumoniae* (17-1692), and New Delhi metallo-β-lactamase 1 (NDM-1+) *K. pneumoniae* (14-3335). The minimum inhibitory concentrations (MIC) of the extracts and the positive control levofloxacin were determined in duplicate by the micro-dilution broth method in 96-well microplates [51]. The aqueous extract was dissolved in distilled water, while organic extracts and levofloxacin were dissolved in dimethyl sulfoxide (DMSO) (Baker, Phillipsburg, New Jersey, USA). The solutions were then diluted in Mueller-Hinton broth (Difco, Detroit, MI, USA) in order to achieve concentrations ranging from 500, 250, 125, 62.5, 31.2, 15.6 and 7.8 µg/mL for extracts and 200, 100, 50, 25, 12.5, 6.25 and 3.12 µg/mL for levofloxacin according to the literature [25]. The range of concentrations used for DMSO was from 6% to 0.09% (*v*/*v*) and this solution was used as a negative control. The strains were inoculated on plates prepared with 5% blood agar and cultured for 24 h at 37 °C. The strains of *P. aeruginosa and S. epidermidis* were incubated for 48 h at 37 °C. One to three colonies from the blood agar plate were selected and transferred to a tube containing 5 mL of sterile saline solution. The suspension was adjusted to 0.5 MacFarland’s standard (1.5 × 10^8^ CFU). Then, 10 µL of this suspension was transferred into 11 mL Mueller Hinton broth to achieve 1.5 × 10^5^ CFU/mL. One hundred microliter of Mueller Hinton broth was added into each well of the 96-well plate. Further, 100 µL of each solution to be tested was added to the wells of line A. Then, a serial dilution (1:2) was carried out through the plate until line G. Then, 100 µL of bacterial suspension (1.5 × 10^8^ CFU) was added to all the wells except line H which was the sterility control. Plates were incubated at 37 °C for 24 or 48 h depending on the bacteria. After the incubation, the turbidity or bottom deposition was visually evaluated to determine the microorganism viability. The MIC values were determined as the lowest concentration able to inhibit the microorganism growth. According with the National Committee for Clinical Laboratory Standards, extracts with a MIC value ≥30 µg/mL were considered negative for antibacterial activity [25].

### 4.5. Cytotoxic Activity

The cytotoxic activity was investigated on human cancer cell lines PC3 (prostate cells), Hep3B (liver cells), HepG2 (liver cells), MCF7 (breast cells), A549 (lung cells) and HeLa (cervical cells) obtained from the ATCC. PC3 cells were cultured in medium RPMI-1640 (Sigma-Aldrich, St. Louis, MO, USA), and the other cells in medium DMEM (Sigma-Aldrich, St. Louis, MO, USA), supplemented with fetal bovine serum 10% (Gibco, Gaithersburg, MD, USA), 2 mM glutamine, and incubated at 37 °C in an atmosphere of 5% CO_2_. Cell passages were maintained in T75 flasks and passages 4-15 were used for the experiments. Prior to treatments, cells were dissociated with TrypLE Express (Gibco, Gaithersburg, MD, USA), seeded at approximately 5000 cells per well in the 96-well plate and allowed to adhere overnight. Cell count and viability were determined using Neubauer hemocytometer and trypan blue staining. The concentrations used for the extracts and for the positive control paclitaxel were 100, 10, 1, 0.1, 0.001 µg/mL for a dose/response with an exposition of 72 h, according to recommendations [26]. Thus, concentrations assayed allowed the determination of the half maximal inhibitory concentration (IC_50_) by a regression analysis with the statistical program Prism 5. The guideline used as reference was the National Cancer Institute, which considers cytotoxic the extracts with an IC_50_ ≤ 30 µg/mL [26]. The proliferation was determined using the CellTiter 96 Assay kit (Promega, Madison, WI, USA), following the manufacturer’s protocol. The absorbance was quantified at 450 nm using an ELISA reader. The experiments were performed in triplicate in three independent experiments.

## 5. Conclusions

This is the first report of the qualitative metabolic profile of *C. trifoliata* and its antibacterial and cytotoxic evaluation. The extracts of *C. trifoliata* stems were rich in terpenes, flavonoids and stilbenes. The hexane and aqueous extracts showed cytotoxic activity in vitro against Hep3B, HepG2 and MCF7 cancer cells. Overall, this study suggests that the cytotoxic activity can be partially explained by their metabolic profile rich in bioactive compounds. This work provides evidence of the anticancer effects of this plant used in the traditional medicine and justify further study of the antitumor activities *C. trifoliata*.

## Figures and Tables

**Table 1:** GC-MS analysis of hexane stems extract of *C. trifoliata*.

RT (min)	Abundance (%)	Molecular Weight	Molecular Formula	Tentatively Identified Compound	Retention Index	Metabolite Class
66.472	14.39	256.4241	C_16_H_32_O_2_	Hexadecanoic acid	1964	Fatty acid
66.623	5.35	284.4772	C_18_H_36_O_2_	Hexadecanoic acid ethyl ester	1994	Fatty ester
74.039	12.60	280.4455	C_18_H_32_O_2_	9*Z*,12Z-Octadecadienoic acid	1977	Fatty acid
74.328	4.63	282.4614	C_18_H_34_O_2_	9*Z*-Octadecenoic acid	2140	Fatty acid
75.294	4.42	284.4772	C_18_H_36_O_2_	Octadecanoic acid	2188	Fatty acid
83.176	2.01	312.5304	C_20_H_40_O_2_	Eicosanoic acid	2366	Fatty acid
89.609	1.94	394.7601	C_28_H_58_	Octacosane	2800	Alkane
95.271	3.15	410.7180	C_30_H_50_	Squalene	2847	Triterpene
102.377	10.45	408.7867	C_29_H_60_	Nonacosane	2900	Alkane
108.871	12.82	436.8399	C_31_H_64_	Hentriacontane	3100	Alkane
111.546	1.81	400.6801	C_28_H_48_O	Campesterol	3131	Sterol
112.571	1.91	412.6908	C_29_H_48_O	Stigmasterol	3170	Sterol
113.143	1.73	454.4749	C_30_H_62_O_2_	1,30-Triacontanediol	3241	Alcohol
114.588	11.23	414.7067	C_29_H_50_O	β-sitosterol	3187	Sterol
116.401	6.53	426.7174	C_30_H_50_O	Lupeol	3320	Triterpene
118.246	5.03	412.6908	C_29_H_48_O	Stigmast-4-en-3-one	3435	Ketone

**Table 2:** UPLC-QTOF-MS analysis of CHCl_3_-MeOH stems extract of *C. trifoliata*.

RT (min)	Experimental *m*/*z* [M–H]^−^	Theoretical Mass	Mass Error (ppm)	Molecular Formula	Tentatively Identified Compound	Metabolite Class
0.612	593.1497	594.1590	1.69	C_27_H_30_O_15_	Kaempferol-O-α-rhamnosyl-glucopyranoside	Flavonoid
2.419	625.1436	626.1488	1.60	C_27_H_30_O_17_	Myricetin 3-O-rutinoside	Flavonoid
2.857	507.1147	508.1222	1.98	C_23_H_24_O_13_	Syringetin 3-*O*-galactoside	Flavonoid
3.226	405.1198	406.1269	2.47	C_20_H_22_O_9_	Piceatannol glucoside	Stilbene
3.547	595.1341	596.1382	1.68	C_26_H_28_O_16_	Quercetin 3-O-glucosyl-xyloside	Flavonoid
3.774	310.2052	-	-	-	Unknown	-
4.042	315.0717	316.0799	3.18	C_13_H_16_O_9_	Protocatechuic acid hexoside	Phenolic
4.807	433.1140	434.1218	2.32	C_21_H_22_O_10_	Dihydrokaempferol 3-*O*-rhamnoside	Flavonoid
5.090	389.1249	390.1320	2.58	C_20_H_22_O_8_	Resveratrol 3-O-glucoside	Stilbene
5.813	473.0362	474.0439	2.12	C_21_H_14_O_13_	Trigallic acid	Phenolic
5.895	431.0939	-	-	-	Unknown	-
6.180	335.0403	336.0486	3.00	C_15_H_12_O_9_	Methyl digallate	Phenolic
6.423	433.0760	434.0854	2.32	C_20_H_18_O_11_	Quercetin arabinoside	Flavonoid
6.531	336.1840	-	-	-	Unknown	-
6.592	615.1869	616.1950	1.63	C_34_H_32_O_11_	Pallidol-3-*O*-glucoside	Stilbene
6.763	447.0938	448.1011	2.24	C_21_H_20_O_11_	Kaempferol 3-O-galactoside	Flavonoid
7.169	615.0988	616.1069	1.63	C_28_H_24_O_16_	Myricitrin O-gallate	Flavonoid
7.191	297.3810	-	-	-	Unknown	-
7.417	253.2161	254.2251	3.96	C_16_H_30_O_2_	Hexadecenoic acid	Fatty acid
7.534	279.2348	280.2407	3.58	C_18_H_32_O_2_	Octadecadienoic acid	Fatty acid
7.595	255.2345	256.2407	3.92	C_16_H_32_O_2_	Palmitic acid	Fatty acid
7.852	283.2649	284.2720	3.54	C_18_H_36_O_2_	Stearic acid	Fatty acid
8.272	653.2235	-	-	-	Unknown	-
9.480	535.1650	-	-	-	Unknown	-

**Table 3:** UPLC-QTOF-MS analysis of aqueous stems extract of *C. trifoliata*.

RT (min)	Experimental *m*/*z* [M–H]^−^	Theoretical Mass	Mass Error (ppm)	Molecular Formula	Tentatively Identified Compound	Metabolite Class
0.612	592.9786	594.1590	1.98	C_27_H_30_O_15_	Apigenin-6,8-di-C- glycoside	Flavonoid
2.781	563.0218	564.1484	1.99	C_26_H_28_O_14_	Kaempferol rhamnosyl xyloside	Flavonoid
3.180	405.1198	406.1269	2.47	C_20_H_22_O_9_	Piceatannol glucoside	Stilbene
3.497	595.1341	596.1382	1.68	C_26_H_28_O_16_	Quercetin 3-O-glucosyl-xyloside	Flavonoid
3.689	609.1451	610.1539	1.65	C_27_H_30_O_16_	Kaempferol 3,7-O-diglucoside	Flavonoid
4.457	374.4914	-	-	-	Unknown	-
4.665	593.1497	594.1590	1.69	C_27_H_30_O_15_	Kaempferol-O-α-rhamnosyl-glucopyranoside	Flavonoid
5.078	453.1356	454.1421	2.21	C_28_H_22_O_6_	*E*-Viniferin	Stilbene
5.395	400.3705	-	-	-	Unknown	-
5.973	755.2030	756.2118	1.33	C_33_H_40_O_20_	Kaempferol 3-O-glucosyl-rhamnosyl-galactoside	Flavonoid
6.179	594.1627	-	-	-	Unknown	-
6.423	433.0760	434.0854	2.32	C_20_H_18_O_11_	Quercetin arabinoside	Flavonoid
6.779	448.1011	449.1089	2.24	C_21_H_21_O_11_	Cyanidin 3-O-galactoside	Flavonoid
6.954	464.0960	465.1038	2.16	C_21_H_21_O_12_	Delphinidin 3-O-glucoside	Flavonoid
7. 384	447.0930	448.1011	2.24	C_21_H_20_O_11_	Kaempferol hexoside	Flavonoid
7.465	576.4380	-	-	-	Unknown	-
7.645	302.0060	-	-	-	Unknown	-
7.851	426.7290	-	-	-	Unknown	-

**Table 4:** Activity of the extracts of *Cissus trifoliata* stems and levofloxacin against bacteria (µg/mL).

Bacteria	Hexane	CHCl_3_-MeOH	Aqueous	Levofloxacin
*S. aureus* (ATCC, 29213)	≥500	≥500	≥500	3.12
*S. epidermidis* (ATCC, 14990)	≥500	≥500	≥500	3.12
*E. faecium* (ATCC, 2127)	≥500	≥500	≥500	3.12
*E. coli* (ATCC, 25922)	≥500	≥500	≥500	3.12
*P. aeruginosa* (ATCC, 27853)	≥500	≥500	≥500	3.12
*K. pneumoniae* (ATCC, 19606)	≥500	≥500	≥500	3.12
*A. baumanni* (ATCC, 13883)	≥500	≥500	≥500	3.12
Methicillin-resistant *S.aureus* (14-2095)	≥500	≥500	≥500	12.5
Linezolid-resistant *S. epidermidis* (14-583)	≥500	≥500	≥500	6.25
Vancomycin-resistant *E. faecium* (10-984)	≥500	≥500	≥500	12.5
ESBL- resistant *E.coli* (14-2081)	≥500	≥500	≥500	25.0
Carbapenem-resistant *P. aeruginosa* (13-1391)	≥500	≥500	≥500	12.5
Oxacillin-resistant *K. pneumoniae* (17-1692)	≥500	≥500	≥500	6.25
NDM-1+- resistant *K. pneumoniae* (14-3335)	≥500	≥500	≥500	50.0
Carbapenem-resistant *A. baumannii* (12-666)	≥500	≥500	≥500	12.5

ESBL: Extended spectrum β-lactamase; NDM-1+: New Delhi metallo-β-lactamase.

**Table 5:** Activity of *C. trifoliata* stem extracts against cancer cell lines.

Cell line	Hexane	CHCl_3_-MeOH	Aqueous	Paclitaxel
HepG2	26 ± 2	80 ± 8	79 ± 5	64.0 × 10^−3^
Hep3B	24 ± 2	81 ± 4	81 ± 7	33.0 × 10^−3^
MCF7	30 ± 3	78 ± 5	30 ± 2	5.12 × 10^−3^
HeLa	35 ± 3	82 ± 4	90 ± 8	5.12 × 10^−3^
A549	51 ± 4	85 ± 3	94 ± 6	4.27 × 10^−3^
PC3	62 ± 3	61 ± 3	58 ± 4	79.4 × 10^−3^

The IC_50_ µg/mL was determined by MTS and is showed as mean ± SD.

## Abbreviations

A431	Human epidermoid carcinoma in cell line
ATCC	American Type Culture Collection
Bcl2	B-cell lymphoma 2
CaCo-2	Human colon caucasian colon adenocarcinoma
CDKs	Cyclin-dependent kinases
CFU	Colony forming units
ER	Estrogen receptor
ESI	Electrospray ionization
GC	Gas Chromatography
HeLa	Human cervix adenocarcinoma cell line
Hep3B	Human Hepatocellular Carcinoma cell line
HepG2	Human Hepatocellular Carcinoma cell line
HMDB	Human Metabolome Database
LC	Liquid chromatography
MCF7	Human breast carcinoma cell line
MIC	Minimum Inhibitory Concentration
MS	Mass spectrometry
MTS	[3-(4,5-dimethylthiazol-2-yl)-5-(3-carboxymethoxyphenyl)-2-(4-sulfophenyl)-2H-tetrazolium
NDM-1	New Delhi metallo-beta-lactamase
NIST	National Institute Standard and Technology
p21	Cyclin-dependent kinase inhibitor
PC3	Human prostate cancer cell line.
QTOF	Quadrupole Time of Flight
UPLC	Ultra High-Performance Liquid Chromatography
